# Tryptophan-like and humic-like fluorophores are extracellular in groundwater: implications as real-time faecal indicators

**DOI:** 10.1038/s41598-020-72258-2

**Published:** 2020-09-21

**Authors:** James P. R. Sorensen, Andrew F. Carr, Jacintha Nayebare, Djim M. L. Diongue, Abdoulaye Pouye, Raphaëlle Roffo, Gloria Gwengweya, Jade S. T. Ward, Japhet Kanoti, Joseph Okotto-Okotto, Laura van der Marel, Lena Ciric, Seynabou C. Faye, Cheikh B. Gaye, Timothy Goodall, Robinah Kulabako, Daniel J. Lapworth, Alan M. MacDonald, Maurice Monjerezi, Daniel Olago, Michael Owor, Daniel S. Read, Richard G. Taylor

**Affiliations:** 1 British Geological Survey, Maclean Building, Wallingford, OX10 8BB UK; 2 Department of Geography, University College London, London, WC1E 6BT UK; 3 Department of Geology and Petroleum Studies, Makerere University, Kampala, Uganda; 4 Department of Geology, Universite Cheikh Anta Diop, Dakar, Senegal; 5 Chancellor College, University of Malawi, P.O. Box 280, Zomba, Malawi; 6 British Geological Survey, Keyworth, NG12 5GG UK; 7 Department of Civil and Environmental Engineering, University of Surrey, Guildford, GU2 7XH UK; 8 Department of Geology, University of Nairobi, Nairobi, Kenya; 9 Victoria Institute for Research on Environment and Development (VIRED) International, Rabuour Environment and Development Centre, Kisumu-Nairobi Road, P.O. Box, Kisumu, 6423-40103 Kenya; 10 Department of Civil, Environmental and Geomatic Engineering, University College London, London, WC1E 6BT UK; 11 Centre for Ecology and Hydrology, Maclean Building, Wallingford, OX10 8BB UK; 12 Department of Civil and Environmental Engineering, Makerere University, Kampala, Uganda; 13 British Geological Survey, Lyell Centre, Research Avenue South, Edinburgh, EH14 4AP UK

**Keywords:** Environmental sciences, Hydrology

## Abstract

Fluorescent natural organic matter at tryptophan-like (TLF) and humic-like fluorescence (HLF) peaks is associated with the presence and enumeration of faecal indicator bacteria in groundwater. We hypothesise, however, that it is predominantly extracellular material that fluoresces at these wavelengths, not bacterial cells. We quantified total (unfiltered) and extracellular (filtered at < 0.22 µm) TLF and HLF in 140 groundwater sources across a range of urban population densities in Kenya, Malawi, Senegal, and Uganda. Where changes in fluorescence occurred following filtration they were correlated with potential controlling variables. A significant reduction in TLF following filtration (ΔTLF) was observed across the entire dataset, although the majority of the signal remained and thus considered extracellular (median 96.9%). ΔTLF was only significant in more urbanised study areas where TLF was greatest. Beneath Dakar, Senegal, ΔTLF was significantly correlated to total bacterial cells (ρ_s_ 0.51). No significant change in HLF following filtration across all data indicates these fluorophores are extracellular. Our results suggest that TLF and HLF are more mobile than faecal indicator bacteria and larger pathogens in groundwater, as the predominantly extracellular fluorophores are less prone to straining. Consequently, TLF/HLF are more precautionary indicators of microbial risks than faecal indicator bacteria in groundwater-derived drinking water.

## Introduction

Fluorescence spectroscopy is a rapid, reagentless technique used to characterise fluorescent natural organic matter (OM) in water^1–7^. There is substantial evidence that natural waters contaminated with wastewater display enhanced fluorescent OM^8–14^. This property led to the suggestion that fluorescence spectroscopy could be an early-warning indicator for the wastewater contamination of drinking water^15^.

Multiple studies have now demonstrated strong associations between the presence and enumeration of thermotolerant coliform (TTCs), or specifically *Escherichia coli*, and fluorescent OM peaks in groundwater-derived drinking water^16–22^. The associated OM peaks are tryptophan-like fluorescence (TLF) and humic-like fluorescence (HLF) that occur at excitation/emission wavelengths pairs of 280/350 and 320–360/400–480 nm, respectively, and can be quantified instantaneously in-situ with portable fluorimeters.

A wide range of studies demonstrating relationships between TLF/HLF and TTCs may indicate fluorescence is emitted by fluorophores within bacteria cells. Indeed, Fox et al.^23^ noted that at least 75% of TLF produced from *E. coli* cells cultured in the laboratory was intracellular in nature. However, *E. coli* is the favoured organism for the industrial production of tryptophan^24^ and can also excrete indole that also fluoresces within the TLF region^22,25^.

In an assortment of surface waters, Baker et al.^26^ showed 32–86% of TLF was lost following filtration through a 0.2 µm membrane indicating a substantial proportion was associated with particulate and cellular material. They also suggested HLF was mainly as dissolved humic material and showed little change following filtration with 70–96% of the signal remaining after filtration. Samples from the River Leith, NW England, in an area featuring groundwater–surface interaction showed a slightly lower TLF loss following filtration of 20–40%^27^, albeit through a larger pore-size 0.45 µm membrane that would not remove all microbes. In groundwater, we might expect an even higher proportion of TLF and HLF to be extracellular due to natural filtration during recharge and subsurface flow, as well as, typically, a lower microbial biomass^28–31^. This expectation would have implications for TLF/HLF use as faecal indicators in groundwater, which provides the majority of the global drinking water supply.

We hypothesise that TLF and HLF are primarily extracellular within groundwater. Previously, we undertook a pilot investigation of 30 groundwater supplies in rural India and revealed a median of 86% of TLF was extracellular^20^. We further examine this hypothesis by evaluating the extracellular nature of TLF, as well as HLF for the first time, in a larger groundwater dataset from four countries with contrasting hydrogeological settings and pollution pressures.

## Materials and methods

### Study areas

**Figure 1:**
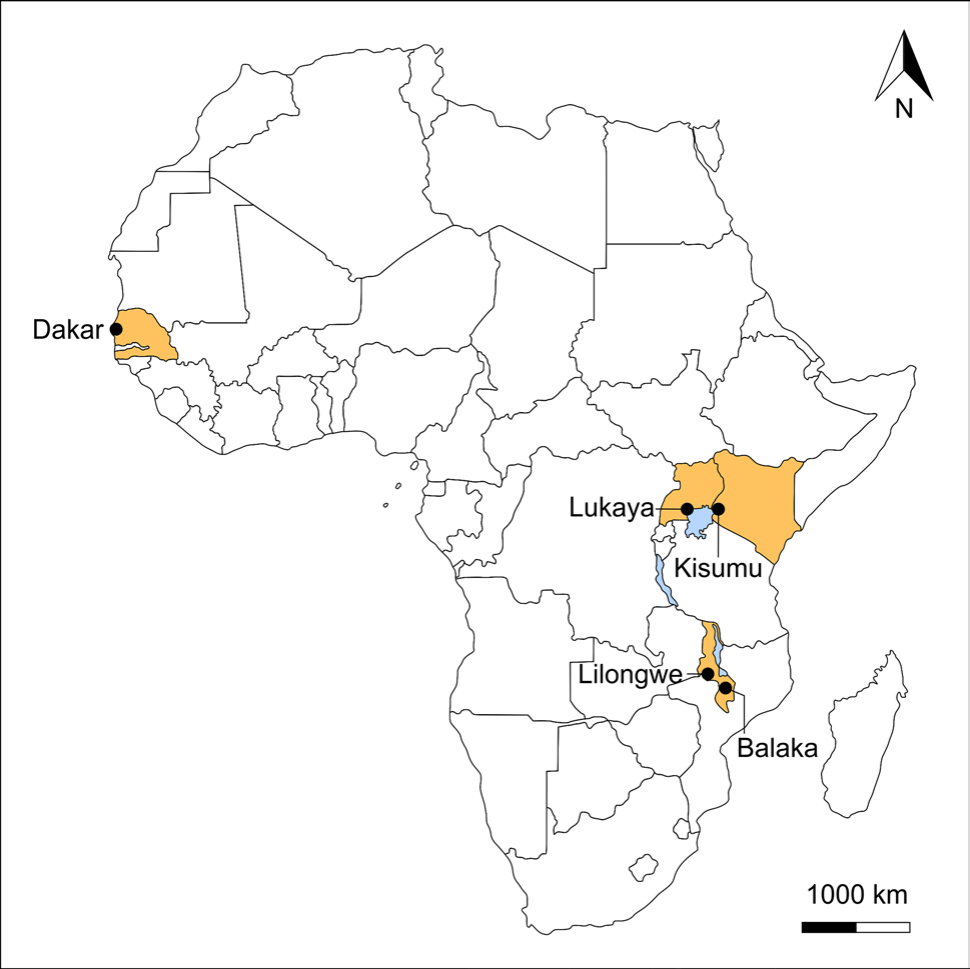
Study site locations in Africa. Continental map modified from https://online.seterra.com/pdf/africa-countries.pdf.

The four study areas together comprise varying degrees of urbanisation from a large city to rural context where pollution sources and pressures vary considerably. Dakar is the large capital city of Senegal with over three million inhabitants constrained within the Cap-Vert peninsular, Kisumu is a medium-sized city of 600,000 residents in Kenya, Lukaya is a small town of around 24,000 people in Uganda, and the rural communities were located in the Lilongwe & Balaka Districts of Malawi (Fig. 1).

The four study areas have contrasting hydrogeological settings. The unconfined shallow Thiaroye aquifer of Dakar comprises Quaternary fine- and medium-grained sands with a shallow water table less than 2 m below ground level (bgl)^32–34^. The heterogeneous volcano-sedimentary Kisumu aquifer system is a suite of Archaean age metasediments, Tertiary volcanics, Quaternary sediments, colluvium and lateritic soils^35–37^. It is multi-layered with unconfined (5–30 m) and confined (> 30 m) horizons. Lukaya predominantly sits on Precambrian Basement with aquifers developed within the weathered overburden and fractured bedrock, in addition to alluvial aquifers towards Lake Victoria. Rest water levels are between 0.5 and 9 m bgl. Lilongwe District is Precambrian Basement with deeper rest water levels between 15 and 25 m bgl^38^. The alluvial aquifers in Balaka District are dominated by clays with significant subordinate sand horizons and a typical rest water level of 5–10 m bgl^38^. Sanitation in all the study areas consists of mainly onsite sanitation^39–42^, which has the potential to faecally contaminate underlying groundwater resources that are used by communities in all settings^20^.

### Groundwater sampling and analysis

A total of 140 groundwater sources were sampled: 29 in Dakar, 38 in Kisumu, 32 in Lukaya, and 41 in Lilongwe & Balaka Districts. Sampling was undertaken in the dry season in Dakar and Kisumu and the wet season in Lukaya and Lilongwe & Balaka Districts. The sources comprised a mixture of pumped boreholes, hand pumped boreholes, open wells, and springs. Samples were obtained from open wells using a 12 V submersible WaSP-P5 pump, except for Lilongwe & Balaka Districts where a rope and bucket were used. Prior to sampling, boreholes, open wells (except Lilongwe & Balaka Districts) and hand pumps flowed for at least 1–2 min to ensure pipework was flushed and the sample was representative of the source. Springs were sampled directly from the outlet: either a discharge pipe in a protected setting or from the surface water channel in an unprotected setting.

TLF was determined using a portable UviLux fluorimeter targeting the excitation–emission peak at λ_ex_ 280 ± 15 nm and λ_em_ 360 ± 27.5 nm (Chelsea Technologies Ltd, UK). HLF in Dakar and Lukaya was quantified by a UviLux fluorimeter configured at an excitation–emission peak at λ_ex_ 280 ± 15 nm and λ_em_ 450 ± 27.5 nm (Chelsea Technologies Ltd, UK). This fluorimeter did not target the centres of the HLF peaks, but was aligned at the same excitation as TLF because of the optical overlap between the TLF and HLF regions. Laboratory calibrations were implemented for all fluorescence measurements, which report quinine sulphate units (QSU) where 1 QSU is equivalent to 1 ppb quinine sulphate dissolved in 0.105 M perchloric acid. Fluorescence analysis was conducted in a HDPE beaker placed within a covered black container to prevent interference from sunlight. Analysis was conducted on unfiltered water to indicate total fluorescence, then passed through a low-protein binding 0.22 µm PVDF membrane (Sterivex, Merck KGaQ, Germany) to sterilise the water and quantify extracellular fluorescence.

Repeatability of TLF fluorimeter data was previously investigated in the laboratory using dissolved tryptophan standards^18^. This study indicated that repeatability was approximately 0.2–0.6 QSU up to 100 QSU, with evidence that absolute repeatability decreased with increasing intensity. To address repeatability in a field situation, including HLF, we calculated 3σ of 74 duplicated measurements in Lukaya. These data ranged between 0–16.5 and 0–49.2 QSU for TLF and HLF, respectively. Repeatability was 0.5 QSU for TLF and 0.3 QSU for HLF.

Specific electric conductivity (SEC), pH, temperature and turbidity were quantified using multiparameter Manta-2 sondes (Eureka Waterprobes, USA) in Dakar, Kisumu, and Lukaya. In Lilongwe & Balaka Districts, temperature, SEC, and turbidity were measured using a HI766EIE1 thermocouple with HI935005 thermometer (Hanna Instruments, USA), S3 portable conductivity meter (METTLER TOLEDO, USA), and 2100Q turbidimeter (Hach Company, USA), respectively.

Thermotolerant coliform (TTC) samples were collected in sterile 250 mL polypropylene bottles and stored in a cool box (up to 8 h) before analysis^18^. TTCs were isolated and enumerated using the membrane filtration method with Membrane Lauryl Sulphate Broth (MLSB, Oxoid Ltd, UK) as the selective medium. Between 0.1 and 100 mL of sample was passed through a 0.45 µm cellulose nitrate membrane (GE Whatman, UK) to ensure colonies were not too numerous to count, whilst maintaining a limit of detection of 1 cfu/100 mL. The membrane was placed on an absorbent pad (Pall Gelman, Germany) saturated with MLSB broth in a plate and incubated at 44 °C for 18–23 h. Plates were examined within 15 min of removal from the incubator and all cream to yellow colonies greater than 1 mm counted as TTCs.

Samples in Dakar and Lukaya for total (planktonic) bacterial cells were collected in 4.5 mL polypropylene cryovials (STARLAB, UK) that were pre-treated with the preservative glutaraldehyde and the surfactant Pluronic F68^43^ at final concentrations of 1% and 0.01%, respectively. The samples were frozen at − 18 °C within 8 h of collection, defrosted overnight during transit to the UK, and analysed the following morning on a BD Accuri C6 flow cytometer equipped with a 488 nm solid state laser (Becton Dickinson UK Ltd, UK). Water samples (500 mL) were stained with SYBR Green I (Sigma-Aldrich, UK) at a final concentration of 0.5% for 20 min in the dark at room temperature, before running on the Accuri at a slow flow rate (14 mL/min, 10 mm core) for 5 min and a detection threshold of 1,500 on channel FL1^21^. A single manually drawn gate was created to discriminate bacterial cells from particulate background, and cells per mL were calculated using the total cell count in 5 min divided by the reported volume run.

### Statistical analyses

The non-parametric paired Wilcoxon test was used to assess the impact of filtration using the null hypothesis that the median difference following filtration is zero^44^. Relationships between change in fluorescence following filtration and various independent variables (total bacterial cells, TTCs, SEC, and turbidity) were assessed using the non-parametric Spearman’s Rank test^45^. Non-parametric techniques were used because of the non-Gaussian distribution of the datasets. All analyses were undertaken in R version 3.4.0 using core commands *wilcox.test* and *cor.test*^46^. Boxplots display the median, the interquartile range, whiskers denote that 10th and 90th percentiles, and dots the 5th and 95th percentiles; these were produced in SigmaPlot version 13.0.

## Results

### Variation in TLF and HLF in the study areas

**Figure 2:**
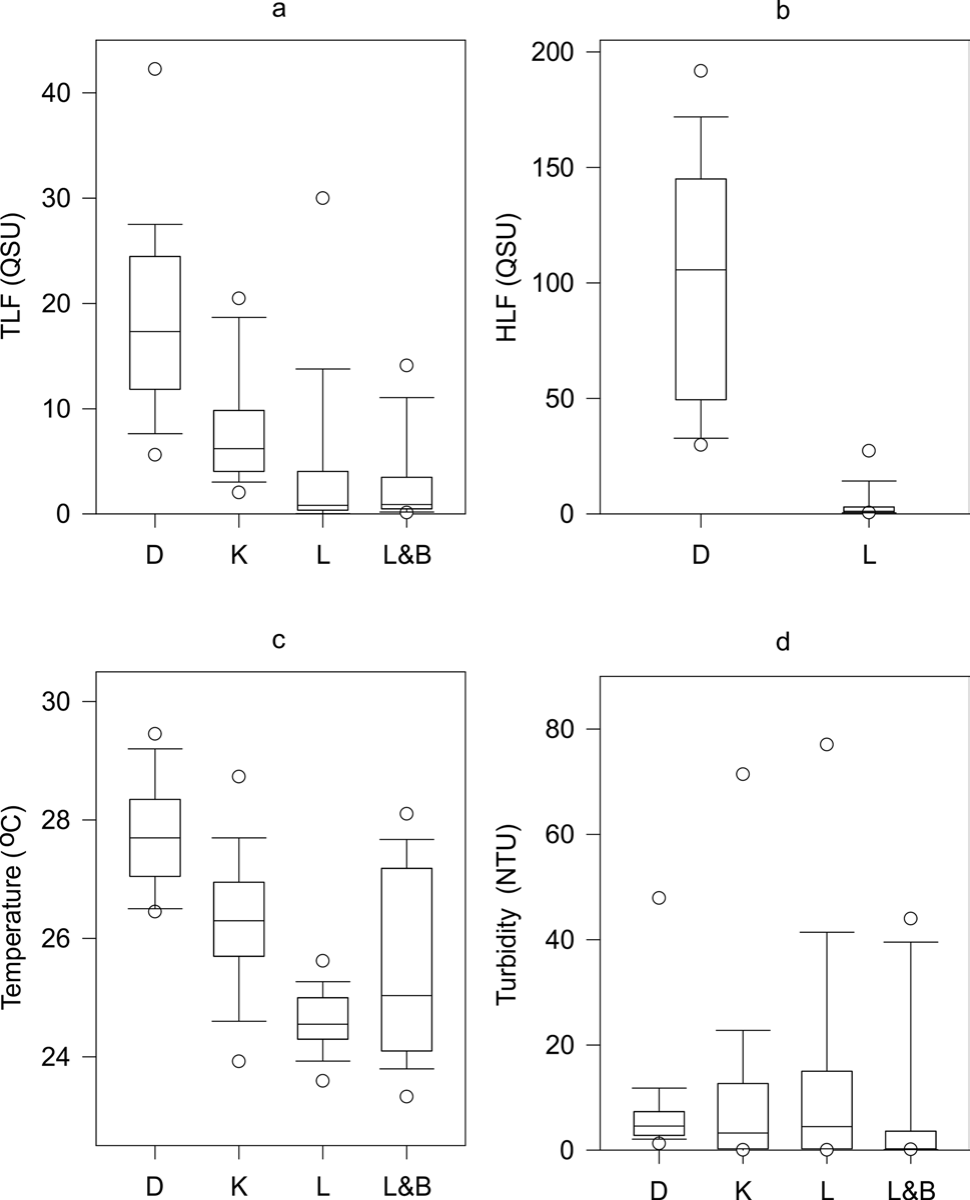
Boxplots of (**a**) unfiltered TLF, (**b**) unfiltered HLF, (**c**) temperature and (**d**) turbidity across D = large city of Dakar, K = medium-sized city of Kisumu, L = small town of Lukaya, and L&B = rural Lilongwe & Balaka Districts.

The intensity of TLF/HLF in groundwater relates to the degree of urbanisation at the land surface (Fig. 2a). Median TLF reduces from 17.4 QSU in the large city of Dakar, to 7.0 QSU in the medium-sized city of Kisumu, and 1.1–1.2 QSU in the small town of Lukaya and rural Lilongwe & Balaka Districts. Furthermore, median HLF in Dakar is almost 90-fold that of Lukaya.

Neither variations in water temperature nor turbidity are considered to appreciably impact the fluorescence results (Fig. 2b–d). Water temperature across all data vary between 23.2 and 29.6 °C and within individual study areas by 2.5–6.0 °C. Therefore, uncertainty relating to temperature is likely to be limited to a maximum of < 6 and < 9% for TLF and HLF, respectively^47–49^. Any optical attenuation relating to suspended solids is also likely to have limited influence on TLF/HLF with a median turbidity of 0.3–4.6 NTU and 97% all of data < 46.2 NTU^50,51^.

### TLF/HLF are predominantly extracellular in groundwater

**Figure 3:**
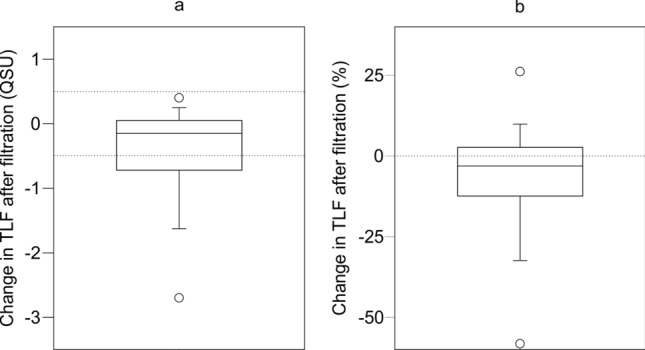
Change in TLF following filtration in (**a**) QSU and (**b**) as a percentage. Dotted lines in (**a**) denote error in repeatability and (**b**) is a reference zero line.

There is a significant change in median TLF following filtration (ΔTLF) across the whole dataset (paired Wilcoxon, *p* < 0.001). Median ΔTLF is a decline of 0.2 QSU (3.1%), which is within the error of repeatability (Fig. 3). The majority (68.6%) of the supplies show no change when considering repeatability uncertainty, with 28.6% declining, and 2.9% increasing. The lower and upper quartiles highlight limited changes of only − 12.2 and 2.6%, respectively.

**Figure 4:**
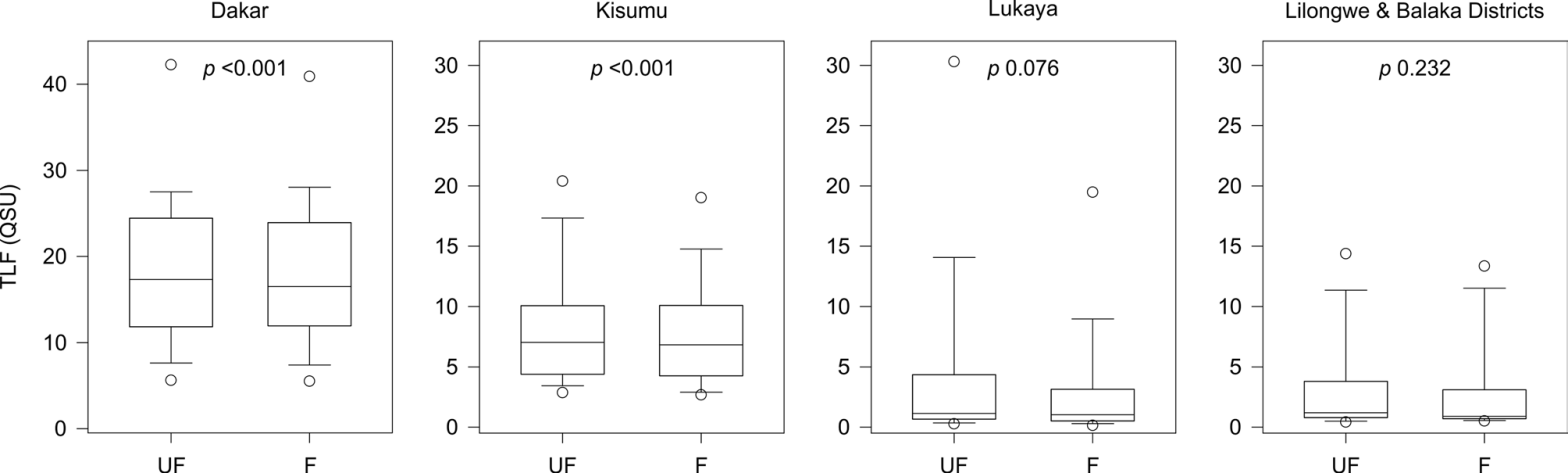
Comparative boxplots of unfiltered (UF) and filtered (F) TLF data for each study area. Displayed *p* values are the results of paired Wilcoxon signed rank tests.

Within the individual country datasets, significant changes following filtration were observed in Dakar and Kisumu, but not Lukaya nor Lilongwe & Balaka Districts (Fig. 4). The lack of a significant change in Lukaya and Lilongwe & Balaka Districts could be a result of the low unfiltered TLF intensities (median 1.1–1.2 QSU). Consequently, any changes following filtration could be harder to detect amongst repeatability uncertainty (± 0.5 QSU). Overall median changes following filtration in Dakar, Kisumu, Lukaya and Lilongwe & Balaka Districts were − 0.4 (− 2.8%), − 0.3 (− 4.2%), − 0.1 (− 6.2%), 0 (0%) QSU, respectively. The greatest loss in TLF following filtration across the entire dataset was at a spring in Lukaya 28.6 QSU (78.6%), with a further spring in the town experiencing a loss of 2.7 QSU (69%). These springs were gentle seepages, and could be considered more representative of slow-moving surface waters; algae were also visible in the channel where samples were obtained. Given any changes following filtration are insignificant or minimal within country datasets collected in both the dry and the wet seasons, TLF is likely to be predominantly extracellular perenially.

**Figure 5:**
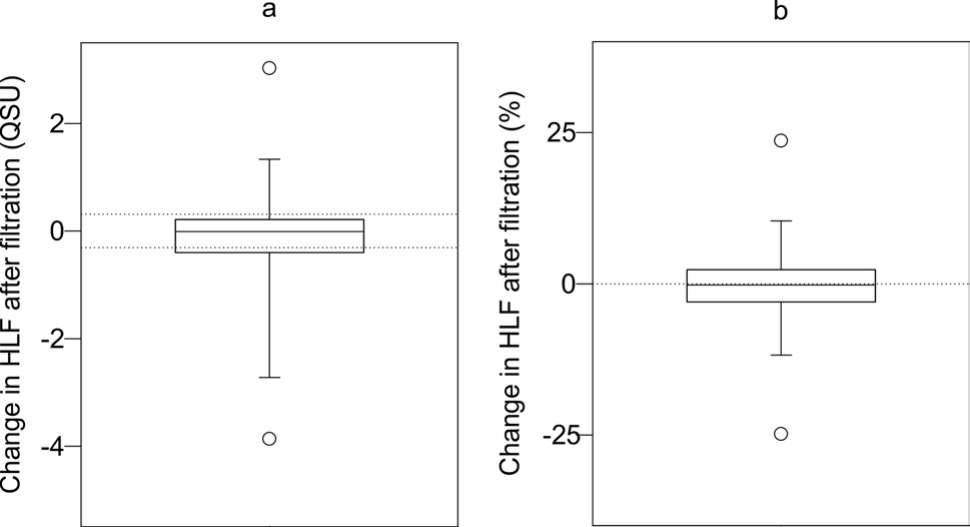
Change in HLF following filtration in (**a**) QSU and (**b**) as a percentage. Dotted lines in (**a**) denote error in repeatability and (**b**) is a reference zero line.

There was no significant change in HLF following filtration (Fig. 5) across the entire dataset (paired Wilcoxon, *p* 0.861), or within both the Dakar (paired Wilcoxon, *p* 0.522) and Lukaya (paired Wilcoxon, *p* 0.704) datasets individually (Figure S1). Although TLF and HLF are correlated in Dakar (ρ_s_ 0.678, *p* < 0.001), contrasting filtration effects suggest that fluorescence is, in part, emanating from two different sources.

### Relationships between TLF change following filtration, total bacterial cells, TTCs, SEC, and turbidity

**Figure 6:**
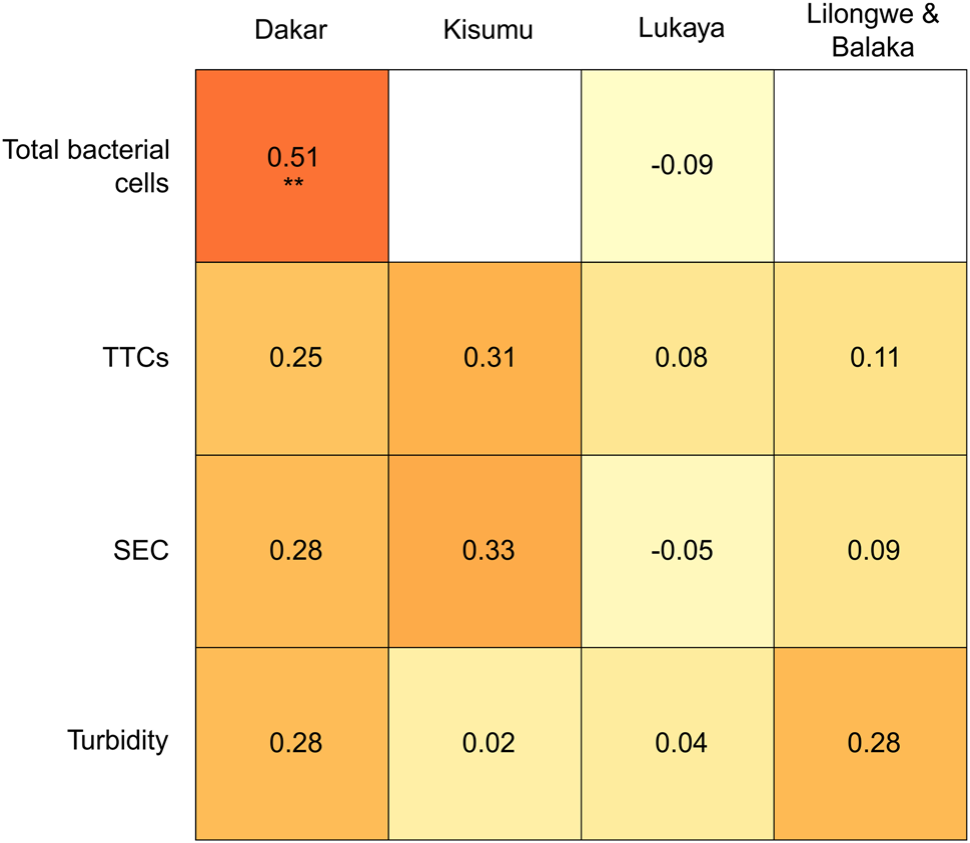
Correlation matrix between four independent variables and TLF change following filtration in each of the four countries. Displayed values are Spearman’s ρ and ** denotes a *p* value of 0.01.

There is a moderate positive correlation between ΔTLF after filtering and total bacterial cells in Dakar (Fig. 6). Note, though, that there is a significant tendency for ΔTLF to decrease with increasing TLF intensity (Figure S2). No other significant correlations exist between ΔTLF and other variables in any country, including turbidity (Fig. 6). It is unsurprising there is no significant correlations in Lukaya and Lilongwe & Balaka Districts, where no significant ΔTLF was observed, but coefficients are included for completeness.

## Discussion

### Implications for TLF/HLF as faecal indicators

TLF and HLF are predominantly extracellular in groundwater, which supports findings from our earlier TLF pilot study in rural India^20^. Extracellular TLF/HLF will have different transport properties to larger faecal indicator bacteria and enteric pathogens, such as bacteria, *Cryptosporidium* oocysts, and *Giardia* that are around 1, 5, and 10 µm, respectively^52^. These organisms will be more readily strained between a faecal source and a groundwater source through both the unsaturated and saturated zones. This behaviour could potentially result in false-positives when organisms are removed completely, whilst an elevated TLF/HLF signal remains. Indeed, false-positives have been highlighted as an issue when defining TLF thresholds to indicate the presence of TTCs^22^. False-positives will be more likely in aquifers exhibiting matrix flow^34^ where faecal indicator bacteria and pathogens are typically restricted to only metres or tens of metres from sources such as pit latrines^53^. Co-transport of TLF/HLF and both TTCs and pathogens is more likely in fractured aquifers where rapid transport of bacteria can occur over several kilometres within a few days, for example Heinz et al.^54^, or alternatively, irrespective of the aquifer, where a source’s integrity is compromised at or near the surface. Co-occurrence of TLF/HLF and TTCs is also more probable where there is a very shallow water table (< 1 m).

Elevated TLF/HLF in the absence of TTCs may still indicate a groundwater source is at-risk. Viruses are the smallest pathogens (27–75 nm) and are often also found in the absence of faecal indicator bacteria and larger pathogens because they can be more mobile in the subsurface^55,56^. Future work could explore TLF as an indicator of virus contamination where faecal indicator bacteria are ineffective. Sorensen et al.^18^ also demonstrated through seasonal sampling that TLF tended to remain perennially elevated in some sources whilst TTCs were more transient. Finally, elevated TLF/HLF is likely to mean a source is better connected to the near surface and potential sources of contamination, even if the fluorescent OM may currently relate to non-faecal sources such as plant litter and soil organic matter.

Our results demonstrate a significant correlation between ΔTLF and total bacterial cells in Dakar, and not between ΔTLF and TTCs. These observations may suggest a proportion of tryptophan-like fluorophores are bound within larger protein molecules within cells, although the relationship could be coincidental and a result of other particulate OM being filtered out. Furthermore, it is supportive of TLF being associated with total microbial biomass^34^ and activity, as opposed to purely TTCs, such as *E. coli*. Previous work has shown a vast assortment of microbes exhibit TLF^57–59^, including ubiquitous species in the environment^23,60,61^.

### Implications for fluorescence sampling

The extracellular nature of TLF/HLF means there are minimal concerns over comparing groundwater fluorescence data collected from in-situ field sensors and samples that have been filtered prior to laboratory analysis. The filtration of laboratory samples is undertaken in many studies to remove suspended solids and sterilise the water to minimise biologically driven OM transformation prior to analysis.

There is no evidence for turbidity attenuating TLF/HLF in groundwater (Fig. 6). Samples with the highest turbidity in each country (46–153 NTU) also showed no evidence of appreciable change in TLF following filtration (1, 2, − 3, 5%, respectively). This result further supports Khamis et al.^50^ in suggesting turbidity is unlikely to have any impact on the in-situ fluorescence monitoring of groundwater. With no observed relationship between ΔTLF and turbidity, and no significant ΔHLF, it is unconfirmed why some ΔTLF/ΔHLF are positive beyond any repeatability error. Many of these positive data are at greater fluorescent intensity, than the repeatability study conducted herein, with previous evidence that error increases with greater intensity; hence, these positives values may not indicate an appreciable change. However, it is also possible some of these data are indicative of either anomalously low unfiltered or high filtered fluorescence values. Anomalous low readings could occur due to air bubbles trapped within the sensor and high readings due to contamination resulting from sensor handling^22^.

## Conclusions

Tryptophan-like fluorophores are predominantly extracellular in groundwater. Significant changes in TLF following filtration were only observed beneath the cities of Kisumu, Kenya, and Dakar, Senegal, where TLF was elevated in comparison to the small town of Lukaya, Uganda and rural Lilongwe & Balaka Districts, Malawi. Nevertheless, TLF was still 93.2–97.2% extracellular on average. In Dakar, tryptophan-like fluorophores associated with the unfiltered > 0.22 µm fraction were moderately correlated with total bacterial cells. Humic-like fluorophores are extracellular in groundwater.

The extracellular nature of TLF/HLF means they will have different transport properties in comparison to faecal indicator bacteria and larger pathogens, which will be more readily strained in both the unsaturated and saturated zones of the subsurface. Co-transport is least likely in intergranular aquifers where the water table exceeds a metre depth below ground level resulting in a higher likelihood of false-positives. TLF/HLF should be considered more precautionary indicators of microbial risks than faecal indicator bacteria in groundwater-derived drinking water.

## Supplementary information

Supplementary Information 1.
