# Supplementary material for: Tryptophan-like and humic-like fluorophores are extracellular in groundwater: implications as real-time faecal indicators
Source: Sci Rep. 2020 Sep 21;10:15379. doi: 10.1038/s41598-020-72258-2 (PMC7505957; doi:10.1038/s41598-020-72258-2)
Supplement: Supplementary file 1 — Supplementary Information 1. [file 41598_2020_72258_MOESM1_ESM.docx]

**Tryptophan-like and humic-like fluorophores are extracellular in groundwater: implications as real-time faecal indicators**

James Sorensen, Andrew F. Carr, Jacintha Nayebare, M. L. Diongue, Abdoulaye Pouye, Raphaëlle Roffo, Gloria Gwengweya, Jade S.T. Ward, Japhet Kanoti, Joseph Okotto-Okotto, Laura van der Marel, Lena Ciric, Seynabou C. Faye, Cheikh B. Gaye, Timothy Goodall, Robinah Kulabako, Daniel J. Lapworth, Alan M. MacDonald, Maurice Monjerezi, Daniel Olago, Michael Owor, Daniel S. Read, Richard G. Taylor


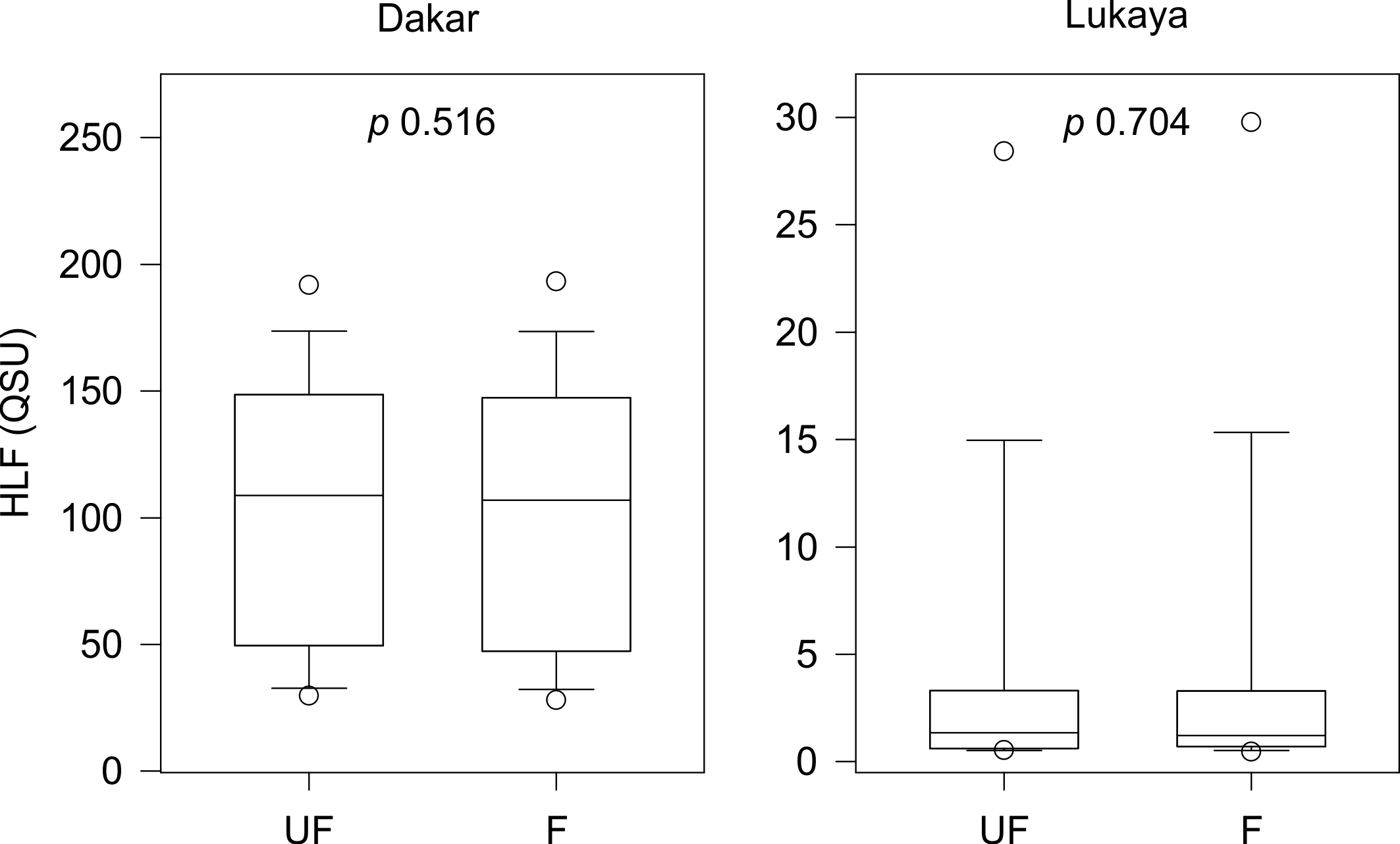


Figure S1 Comparative boxplots of unfiltered (UF) and filtered (F) HLF data for each study area. Displayed p-values are the results of paired Wilcoxon signed rank tests.


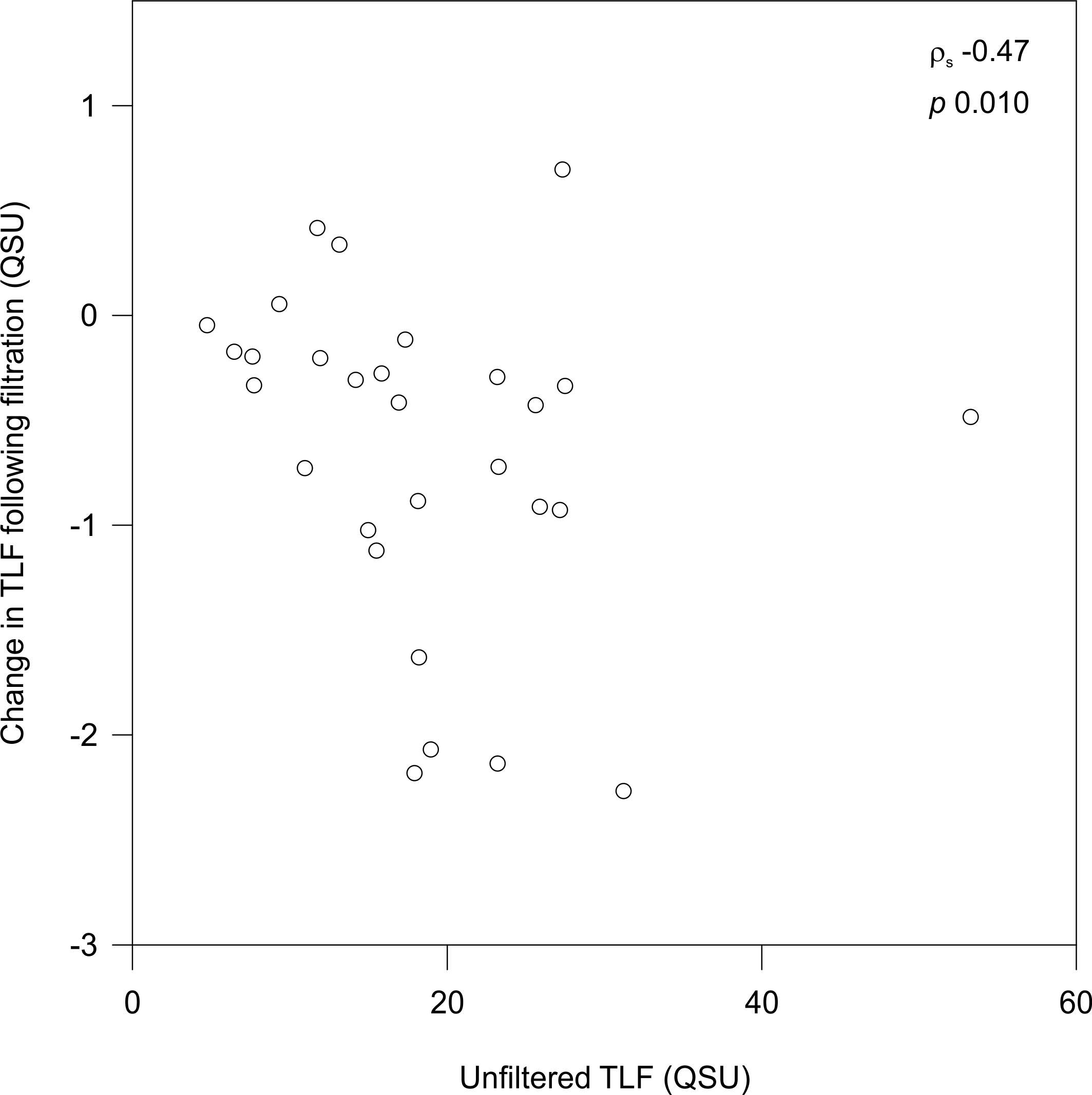


Figure S2 Relationship between unfiltered TLF and change in TLF following filtration beneath Dakar. Spearman’s Rank and associated p-value displayed.
